# Strategies to Address the Lack of Labeled Data for Supervised Machine Learning Training With Electronic Health Records: Case Study for the Extraction of Symptoms From Clinical Notes

**DOI:** 10.2196/32903

**Published:** 2022-03-14

**Authors:** Marie Humbert-Droz, Pritam Mukherjee, Olivier Gevaert

**Affiliations:** 1 Stanford Center for Biomedical Informatics Research Department of Medicine Stanford University Stanford, CA United States; 2 Department of Biomedical Data Science Stanford University Stanford, CA United States

**Keywords:** clinical text mining, weak supervision, text classification, symptom extraction, EHR, machine learning, natural language processing

## Abstract

**Background:**

Automated extraction of symptoms from clinical notes is a challenging task owing to the multidimensional nature of symptom description. The availability of labeled training data is extremely limited owing to the nature of the data containing protected health information. Natural language processing and machine learning to process clinical text for such a task have great potential. However, supervised machine learning requires a great amount of labeled data to train a model, which is at the origin of the main bottleneck in model development.

**Objective:**

The aim of this study is to address the lack of labeled data by proposing 2 alternatives to manual labeling for the generation of training labels for supervised machine learning with English clinical text. We aim to demonstrate that using lower-quality labels for training leads to good classification results.

**Methods:**

We addressed the lack of labels with 2 strategies. The first approach took advantage of the structured part of electronic health records and used diagnosis codes (International Classification of Disease–10th revision) to derive training labels. The second approach used weak supervision and data programming principles to derive training labels. We propose to apply the developed framework to the extraction of symptom information from outpatient visit progress notes of patients with cardiovascular diseases.

**Results:**

We used >500,000 notes for training our classification model with International Classification of Disease–10th revision codes as labels and >800,000 notes for training using labels derived from weak supervision. We show that the dependence between prevalence and recall becomes flat provided a sufficiently large training set is used (>500,000 documents). We further demonstrate that using weak labels for training rather than the electronic health record codes derived from the patient encounter leads to an overall improved recall score (10% improvement, on average). Finally, the external validation of our models shows excellent predictive performance and transferability, with an overall increase of 20% in the recall score.

**Conclusions:**

This work demonstrates the power of using a weak labeling pipeline to annotate and extract symptom mentions in clinical text, with the prospects to facilitate symptom information integration for a downstream clinical task such as clinical decision support.

## Introduction

### Background

Unstructured text from electronic health records (EHR) contains myriads of information that is not encoded in the structured part of EHRs, such as symptoms experienced by the patient. Structuring and managing symptom information is a major challenge for research owing to their complex and multidimensional nature. Extracting symptom information from clinical text is critical; for example, for phenotypic classification, clinical diagnosis, or clinical decision support [1-3]. More specifically, symptoms are crucial to the assessment and monitoring of the general state of the patient [1,4] and are critical indicators of quality of life for chronically ill patients [5,6]. Their evolution through time can be a string indicator of the patient’s clinical status change. Finally, in the context of pandemic prevention, symptoms are used for syndromic surveillance [7,8] and patient characterization [9,10].

Using natural language processing (NLP) and machine learning to process and use clinical text for such applications has great potential [11-14]. Unfortunately, machine learning, and more specifically supervised machine learning, requires a great amount of labeled data to train a model, which is at the origin of the main bottleneck of model development [15]. Manually labeling data sets is extremely costly and time-consuming as multiple experts need to manually review and annotate several hundreds of clinical notes [13,16]. Moreover, the development of such a resource presents unique challenges as the text contains personal information, and access to such data is usually restricted.

Throughout the past years, shared resources such as Informatics for Integrating Biology and the Bedside (i2b2) have generated deidentified and annotated data sets for the development of NLP systems for specific tasks. Such resources remain limited, as most of the annotated data sets contain only hundreds to a few thousands of notes. Moreover, these data sets come from a limited number of institutions, making the development of an NLP system with such data unlikely to generalize to other institutions or other tasks.

To develop NLP systems and models that are transferable between multiple institutions and free of overfitting, a large amount of data needs to be available for training. To do so, alternatives to supervised machine learning have been explored, such as distant supervision, which seeks to include information from existing knowledge bases [17] or active learning, which involves human experts in the machine learning process [18-20]. One method in particular, weak supervision, is attracting increasing attention for the automatic generation of lower-quality labels for unlabeled data sets [21-25].

### Objective

To address the lack of labeled data, we propose 2 alternatives to manual labeling for the generation of training labels for supervised machine learning with clinical text. The first approach takes advantage of the structured part of EHRs and uses diagnosis codes to derive training labels. The second approach uses weak supervision and data programming principles to derive training labels. We propose to apply the developed framework to the extraction of symptom information from outpatient visit progress notes of patients with cardiovascular diseases.

Extracting symptoms from clinical narratives is not a straightforward task as symptoms are often expressed in an abstract manner. A straightforward way of deriving labels from EHR would be to take advantage of their coded part and use the International Classification of Disease–10th revision–Clinical Modification (referred to as ICD-10, henceforth) codes. This approach has challenges, as demonstrated in multiple studies [2,9,26-30]. This is especially true if the target information is symptoms, as the corresponding ICD-10 chapter is typically used when a sign or symptom cannot be associated with a definitive diagnosis. Thus, their occurrence in EHR is very scarce and expected to be incomplete. Despite issues related to inaccuracy in ICD-10 coding, we propose to use such codes to label our training set, with the assumption that with sufficient training data, the poor quality of the labels will be balanced out. Although inaccurate and possibly biased, the use of ICD-10 data is considered standard in many classification studies involving clinical text [15,31-41]. Moreover, we propose to complement the use of ICD-10 codes with a weak supervision approach to derive labels. Weak supervision has gained a great amount of traction in the past years [21-25] as a response to the increased need for training data for machine learning. We used the Snorkel library [42] to combine a large number of clinical reports with noisy labeling functions and unsupervised generative modeling techniques to generate labels for our models. Finally, we test the models on external cohorts as a way to assess the bias and test the generalizability of the models.

We successfully demonstrate that by using a large number of notes for training, we can train a classification model able to recognize specific classes of symptoms using low-quality labels. The resulting model is independent of the prevalence of positive instances and is transferable to a different institution. We show that training our model on such pseudolabels results in a good predictive performance when tested on a data set containing gold labels.

## Methods

### Cohort Description

**Figure 1:**
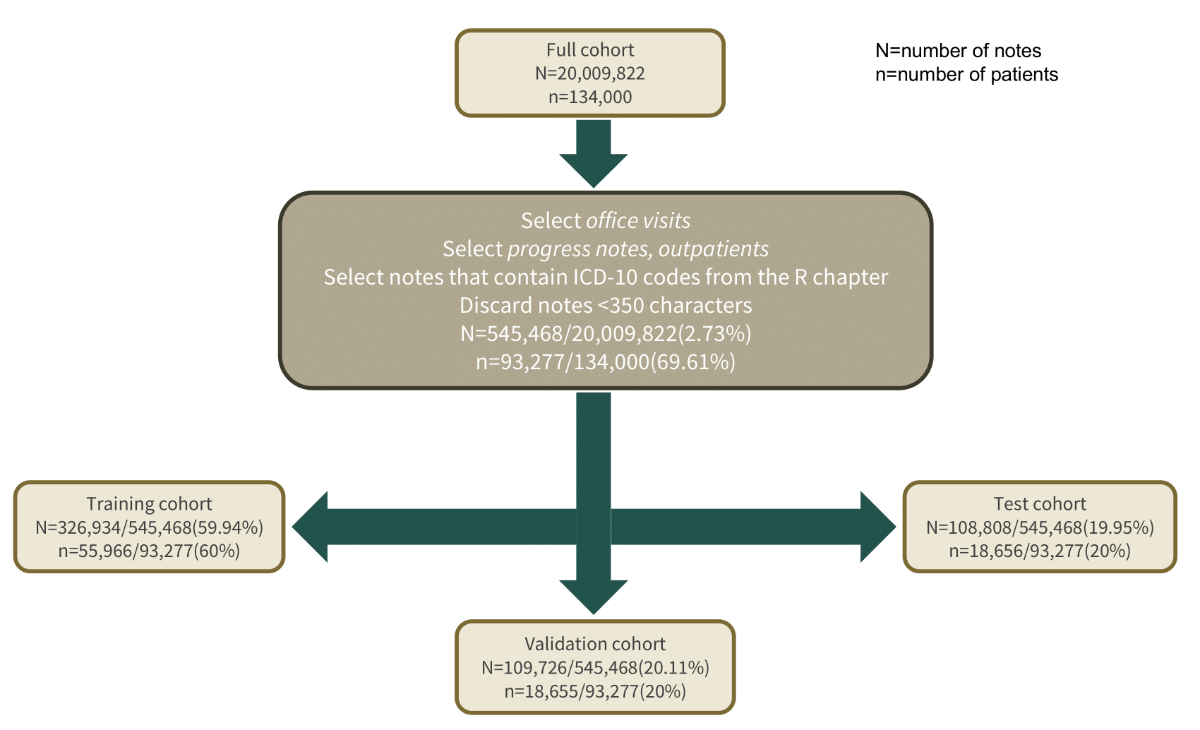
CONSORT (Consolidated Standards of Reporting Trials) diagram for Stanford Health Care–electronic health record symptom extraction. Our full cohort consisted of 20 million notes and 134,000 patients. We selected progress notes from outpatient visits from encounters with International Classification of Disease–10th revision (ICD-10) codes from the chapter R. Notes <350 characters were discarded, yielding 545,468 notes for 93,277 patients.

Our data set consisted of 20,009,822 notes from January 1, 2000, to December 31, 2016, for 134,000 patients with cardiovascular diseases from Stanford Health Care (SHC), collected retrospectively in accordance with the approved institutional review board protocol (IRB-50033) guidelines. Progress notes from outpatient office visits were selected. As the ICD-10 codes for symptoms were chosen for initial labels, encounters without R codes were discarded. Finally, short notes (ie, <350 characters) were also discarded. The final cohort contained 545,468 notes for 93,277 patients (Figure 1).

For prototyping purposes and to evaluate the effect of the training set size on the performance, subsets of the full cohort were created, leading to the following three data set sizes: I (patients: 717/93,277, 0.77%), II (patients: 5611/93,277, 6.02%), and III (patients: 93,277/93,277, 100%). Patients were split into training, validation, and test sets using a 60:20:20 ratio. Table 1 provides a more detailed description of the data sets.

ICD-10 codes describing symptoms and signs involving the circulatory and respiratory systems were used to label the notes for the text classification task. The symptoms considered were only coded at the highest level of the ICD-10 hierarchy. The prevalence of the R codes was low, between 2% and 10% of positive instances (see Table S1 in Multimedia Appendix 1 for details).

**Table 1:** Patient and note distribution for each data set considered in this study.

Data set				I^a^ (N=717)		II^a^ (N=5611)		III (N=93,277)		IV^b^ (93,277)		V^c^ (N=75.692)
Train set, n (%)				430 (59.9)		3360 (59.88)		55,966 (59.99)		55,966 (59.99)		38,381 (50.71)
Validation set, n (%)				143 (19.9)		1123 (20.01)		18,655 (19.99)		18,655 (19.99)		18,655 (24.65)
Test set, n (%)				144 (20.1)		1128 (20.10)		18,656 (20)		18,656 (20)		18,656 (24.65)
Age (years), mean (SD)				60 (23)		58 (23)		59 (23)		59 (23)		53 (23)
**Gender, n (%)**												
		Men	306 (42.7)		2381 (42.43)		51,876 (55.61)		51,876 (55.61)		43,765 (57.82)	
		Women	410 (57.2)		3229 (57.55)		41,396 (44.38)		41,396 (44.38)		31,925 (42.18)	
		Unknown	1 (0.1)		1 (0.02)		5 (0.005)		5 (0.005)		2 (0.003)	
**Total notes, n (%)**				4245 (100)		34,368 (100)		545,468 (100)		871,753 (100)		544,907 (100)
	Train set			2480 (58.42)		20,500 (59.65)		326,934 (59.94)		653,219 (74.93)		326,373 (59.89)
	Validation set			704 (16.58)		6698 (19.49)		109,726 (20.12)		109,726 (12.59)		109,726 (20.14)
	Test set			794 (18.70)		6494 (18.89)		108,808 (19.95)		108,808 (12.48)		108,808 (19.97)

^a^Data sets I and II are subsets of data set III.

^b^Data set IV represents the hybrid data set of labeled and unlabeled notes considered for the weak supervision experiment.

^c^Data set V contains the set of unlabeled notes from IV.

### Pipeline

We defined our task of extracting symptom information from clinical notes as a multi-class classification problem. Machine learning algorithms were trained to classify whether each input note contained a specific class of symptoms.

The proposed pipeline used a subset of the ICD-10 chapter containing symptoms, signs, and abnormal clinical and laboratory findings. The codes in this chapter are typically used when a sign or symptom cannot be associated with a definitive diagnosis. As their occurrence in EHR is expected to be incomplete, we assumed that the presence of a code is associated with the observation of the symptom, but the absence of a code cannot be associated with the absence of the symptom in question.

**Figure 2:**
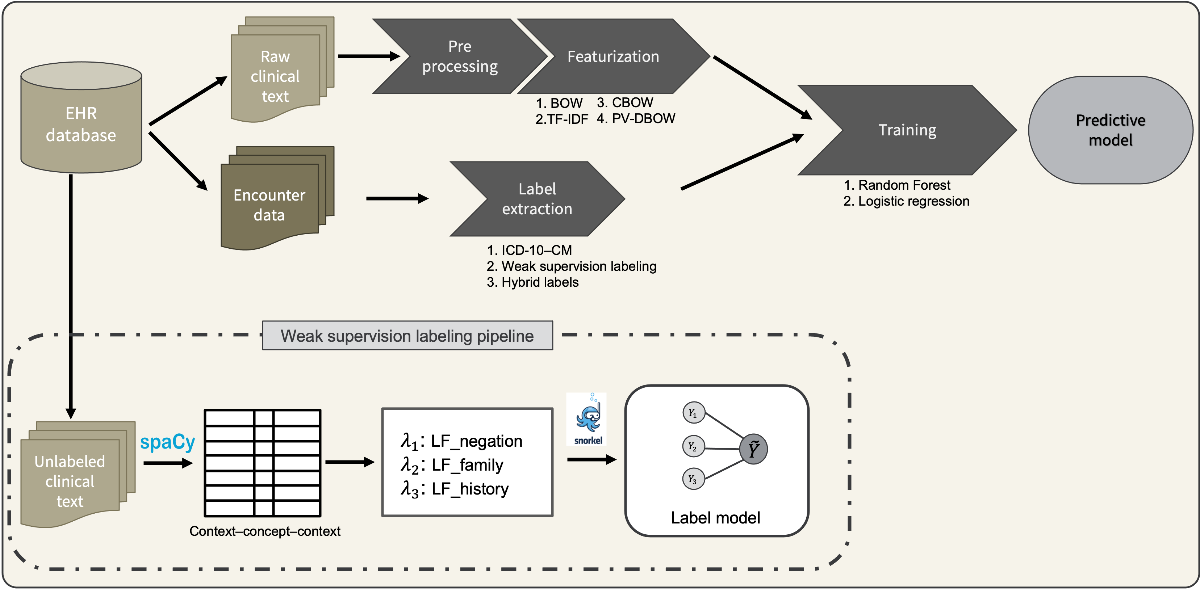
End-to-end pipeline developed for extracting pseudolabels out of an electronic health record (EHR) database and training a text classifier for recognition of presence or absence of symptoms. The approach leverages the structured part of EHR (International Classification of Disease–10th revision–Clinical Modification [ICD-10–CM] codes) and weak supervision to generate labeled training corpus. Three types of labels are used for the training: ICD-10–CM codes; noisy labels obtained by a weak supervision pipeline; and hybrid labels, containing both ICD-10–CM codes and noisy labels. Two machine learning algorithms are considered: random forest and logistic regression. Four featurization methods are considered: bag-of-words (BOW), term frequency–inverse document frequency (TF-IDF), continuous BOW (CBOW), and paragraph vector–distributed BOW (PV-DBOW). LF: labeling function.

The full pipeline developed for this study is depicted in Figure 2. We obtained the raw clinical text and encounter data from the SHC database. The raw text was first preprocessed for standardization purposes. Then, the text was transformed into a numerical format (ie, featurization) so that it can be used as input features for our model training. Then, ICD-10 codes were extracted from the structured encounter data to use as labels. A multi-class classification model was then trained to predict the presence of symptoms in the text. Next, we propose a weak supervision labeling pipeline as an additional method for extracting labels for the downstream prediction task. For that additional part, notes that were initially discarded because of the lack of symptom codes in the encounter data were processed using an entity recognition model with the spaCy library [43] and labeled using a labeling model generated using the Snorkel package [42].

### Preprocessing

To facilitate machine learning techniques, the clinical notes were standardized in the following manner: special characters and numbers were removed; the text was transformed into lower case only; frequent words (eg, the, as, and thus) often denoted as stop words were removed, except negative attributes such as no or not; next, each note was standardized using the Porter stemming algorithm; and finally, the text was tokenized into individual words. Sectioning of the notes was not performed; thus, the entire note was included in the featurization step.

### Featurization

In this report, we evaluated the following approaches for featurization of the clinical notes. The first method, bag-of-words (BOW), is a simple yet effective method to represent text data for machine learning and acts as a baseline. In this method, the frequency of each word is counted, yielding a vector representing the document. As each word represents a dimension of the document vector, the size of the latter is proportional to the size of the vocabulary used. As words are represented by their document frequency, the resulting document vector does not contain any syntactic or contextual information.

Next, we used term frequency–inverse document frequency (TF-IDF), a weighting scheme, in addition to BOW whereby word frequencies from BOW are weighted according to their IDF. This reweighting of the frequencies dampens the effect of extremely frequent or rare words.

Next, we used the continuous BOW (CBOW; also referred to as *word2vec*) algorithm [44]. CBOW is an algorithm that generates word vectors based on a prediction task via a neural network. The output of such a network is an embedding matrix that is used to encode each word into a specific vector. The embedding matrix used in this project was trained on biomedical text (PubMed and Medical Information Mart for Intensive Care–III [MIMIC-III]) by Zhang et al [45]. Word vectors were generated using these pretrained embeddings and then averaged to yield a single document vector representing the entire note. As a result, the document embedding vector was of dimension 200.

Finally, the paragraph vector–distributed BOW (PV-DBOW; also referred to as *doc2vec*) [46], an extension of CBOW to paragraphs, was used to add some syntactic knowledge in the encoding of each document. The vector size for the document was 300 and was independent of the corpus size.

### Weak Labeling

To address the problem of a lack of labels for EHR-based supervised learning, a weak supervision pipeline using the Snorkel package [42] was implemented. Weak supervision allows us to create a set of noisy labels for an unlabeled data set. The noisy labels are generated using a set of *labeling functions*, namely, a set of heuristic rules.

For this project, we implemented labeling functions based on pattern recognition applied to a 20 token–context window (10 tokens before and 10 tokens after the target term) to determine the negation, temporality, and experiencer of the target symptom. We used the publicly available *clinical event recognizer* base terminology [47] to match our context window with negative expressions, historical expressions, and family mentions. If a mention is matched within the context window of a given term, it is labeled accordingly: *absent* if negative expression is matched, *history* if historical expression is matched, and *family* if family mention is matched. Target symptoms that were positive, experienced by the patient, and not part of the past medical history were labeled positive. Occurrences deviating from this pattern were labeled negative.

Symptom recognition was performed using a ScispaCy [48] pipeline trained to recognize biomedical entities. The process of extracting the presence or absence of symptoms belonging to the R00-R09 categories was implemented as follows: the full clinical note is processed with spaCy [43] using the entity recognition model from the ScispaCy library, trained on BioCreative V Chemical Disease Relation corpus, a corpus of 1500 PubMed articles annotated for chemicals, disease, and chemical–disease interactions [49] (en_ner_bc5cdr_md [48]). As we were classifying the notes using only the 3 characters categories of the ICD-10 codes, each entity that was tagged needed to be associated to its corresponding category. For that purpose, we normalized them to the concept unique identifiers from the unified medical language system with the highest similarity score. This allowed us to group each entity to their corresponding ICD-10 category (see Table S2 in Multimedia Appendix 1 for a list of concept unique identifiers). Then, the labeling functions defined earlier were used to generate noisy labels, which can finally be used to train a machine learning model.

### Modeling

The input features were used to predict a set of symptoms related to abnormalities in the circulatory and respiratory systems (ICD-10 codes R00-R09). The problem was approached as a text classification task using a subset of the ICD-10-R codes for the class labels. The classes are not mutually exclusive; therefore, a *one-versus-all* classification was chosen. We compared two classification algorithms for this task, namely random forests [50] and logistic regression [51]. We only report the results obtained with 100 estimators for the random forest and the limited-memory Broyden–Fletcher–GoldfarbShanno solver. The detailed parameters used for each model are provided in the Multimedia Appendix 1.

### Performance Evaluation

We used the following classification metrics to evaluate each model: recall, F1 score, and average precision score. We also computed the receiver operating characteristic (ROC) curves and precision-recall curves. Owing to the class imbalance, we gave more importance to the precision-recall curve. For example, in the case of *hemorrhage from respiratory passages* class of symptoms (R04), the positive instances represent only approximately 1% of the data points. We also considered computation time and memory requirements as important metrics to determine the best classification model. Given the size of our data set, an efficient implementation was of paramount importance for the success of our predictive model.

### External Validation

To assess the impact of training the model on low-quality labels, the models were tested on an external data set developed for symptom extraction by Steinkamp et al [52]. Their work provides an open-source annotated data set for symptom extraction. The notes were 1008 deidentified discharge summaries from the i2b2 2009 Medication Challenge [53]. The set of notes was annotated by 4 independent annotators for all symptom mentions, whether *positive*, *negative,* or *uncertain*. To benchmark our study, we chose three classes of symptoms that were both present in our study and in the annotated data set of Steinkamp et al [52], namely cough (R05), abnormalities of breathing (R06), and pain in throat and chest (R07). As the annotations were performed at the *mention* level but our study was performed at the *note* level, a majority voting algorithm was chosen to assess the note-level polarity of the symptom mention to generate note-level labels. On the basis of the SHC experiments, only models showing the best promise in terms of predictive performance were chosen for this step. More specifically, models trained with the logistic regression algorithm using TF-IDF and PV-DBOW features were chosen for the external validation.

## Results

### Logistic Regression Performs Better Than Random Forest for Predicting the Presence of Symptoms in Outpatient Progress Notes

**Figure 3:**
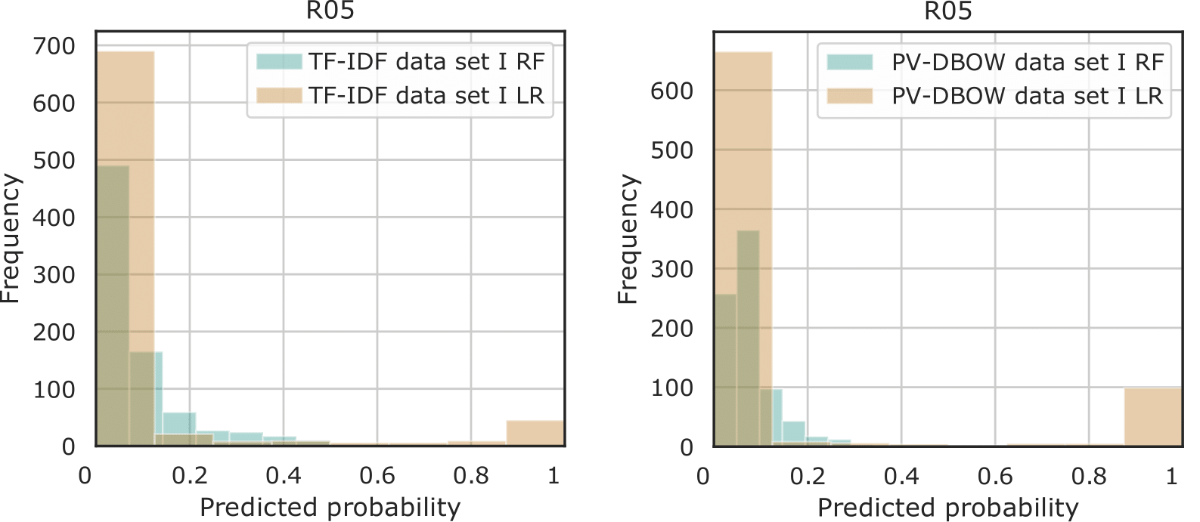
Histogram of predicted probabilities for the presence of the cough symptom (R05) in the outpatient progress note for data set I, with a comparison between probabilities predicted by logistic regression (LR) and random forest (RF) for term frequency–inverse document frequency (TF-IDF) and paragraph vector–distributed bag-of-words (PV-DBOW) feature extraction methods.

**Figure 4:**
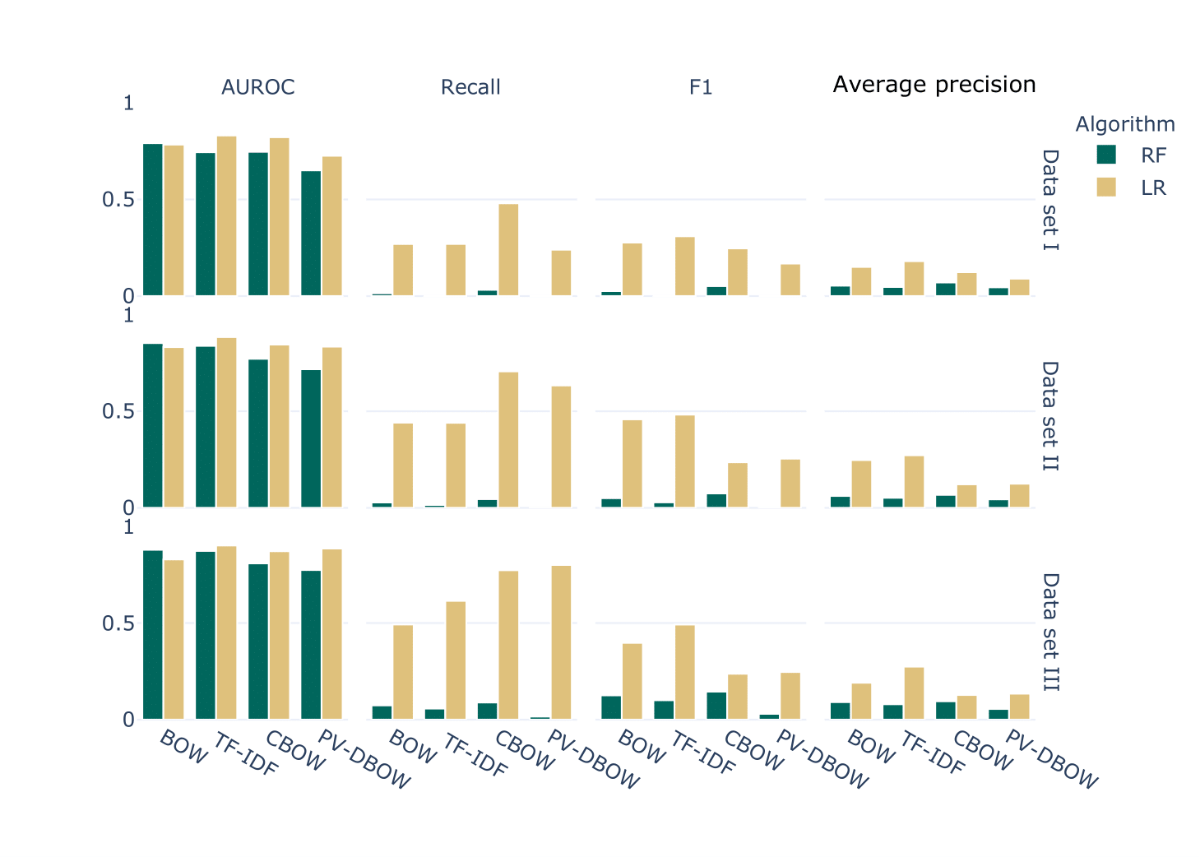
Summary of performance metrics averaged over all codes for all four considered feature extraction methods (bag-of-words [BOW], term frequency–inverse document frequency [TF-IDF], continuous BOW [CBOW], and paragraph vector–distributed BOW [PV-DBOW]). AUROC: area under the receiver operating characteristic curve; LR: logistic regression model; RF: random forest model.

**Figure 5:**
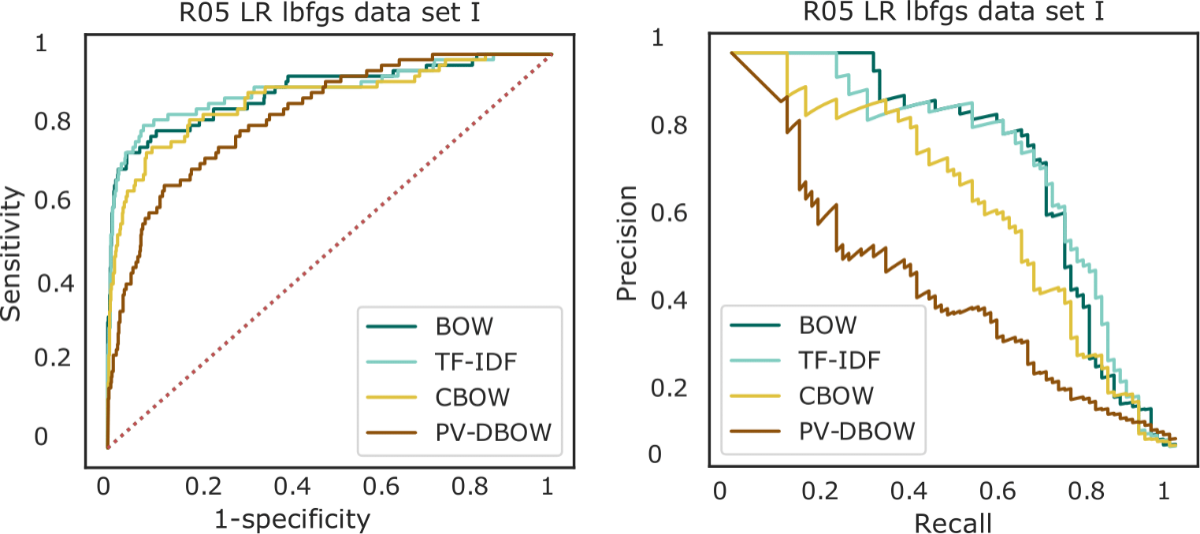
Receiver operating characteristic and precision-recall curves for the prediction on the test set (data set I described in Table 1) of presence of cough (R05) symptoms from outpatient progress notes using logistic regression (LR) with 4 feature extraction methods. BOW: bag-of-words; CBOW: continuous BOW; lbfgs: limited-memory Broyden–Fletcher–GoldfarbShanno solver; PV-BOW: paragraph vector–distributed BOW; TF-IDF: term frequency–inverse document frequency.

Outpatient progress notes collected from January 1, 2000, to December 31, 2016, from the SHC EHR database were used to train a text classifier to extract symptoms related to abnormalities in the circulatory and respiratory systems (Figure 1). Two machine learning algorithms were considered, namely random forest and logistic regression. The models were first built on a subset of the cohort for prototyping purposes (Table 1: data set I). Random forest showed poor predictive performance, with no or few positive instances predicted (Figure 3). Without exception, logistic regression outperformed random forest for all the considered data set sizes (Figure 4). Use of TF-IDF features to predict the presence of symptoms in the notes led to the best overall performance (Figure 5).

### Embedding-Based Methods Perform Better With Increasing Data Set Size

To demonstrate that increasing the size of the training set significantly improves the performance of deep learning–based embedding methods, the classification task was performed on 3 different data set sizes, ranging from 0.75% (700/93,277) of patients to 100% (93,277/93,277) of patients (Table 1).

**Figure 6:**
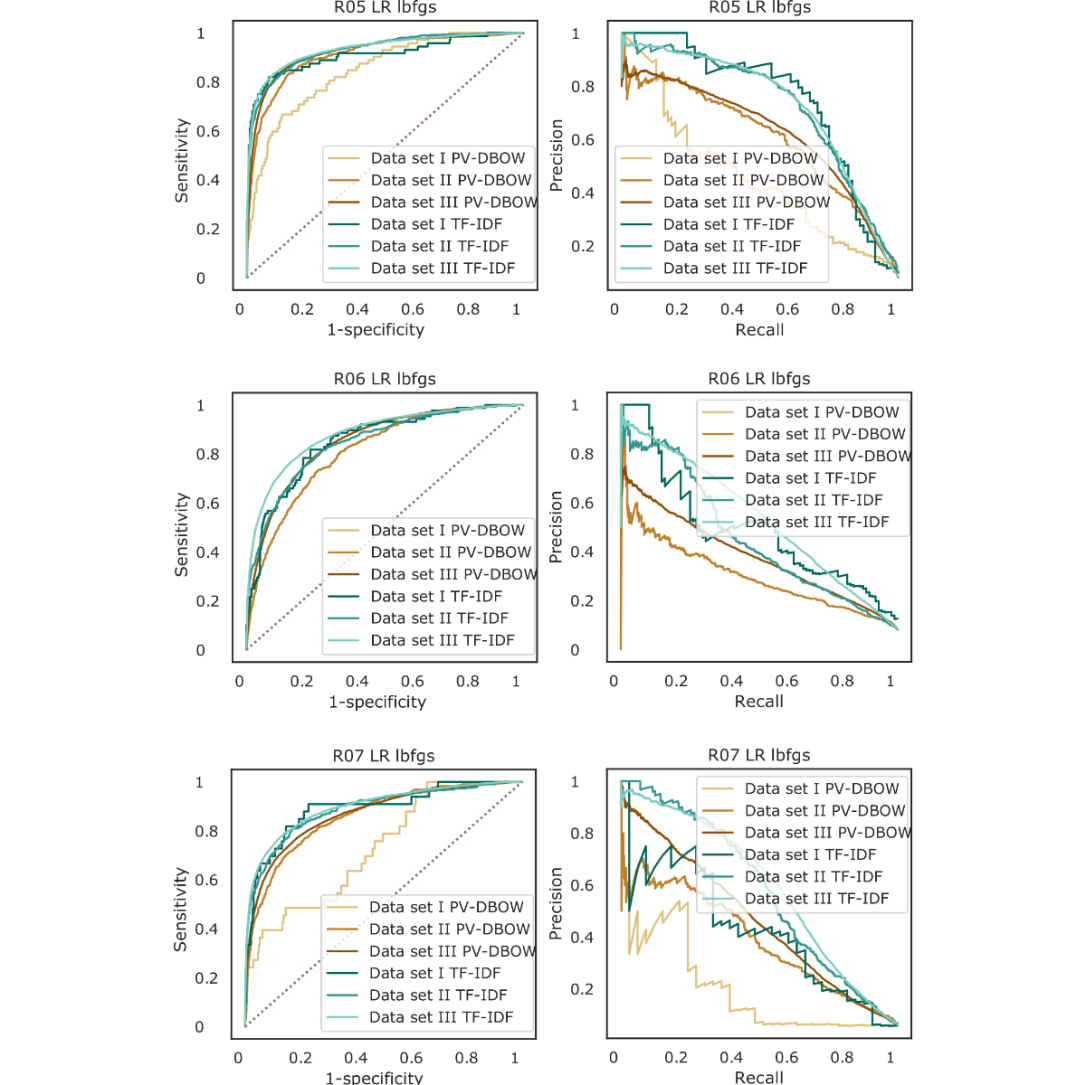
Comparison of receiver operating characteristic (left column) and precision-recall (right column) curves for the prediction of presence of cough (R05), abnormality of breathing (R06), and pain in throat and chest (R07) classes of symptoms from outpatient progress notes using logistic regression (LR) with the limited-memory Broyden–Fletcher–GoldfarbShanno (lbfgs) solver on data set I, data set II and data set III with term frequency–inverse document frequency (TF-IDF) and paragraph vector–distributed bag-of-words (PV-DBOW) features.

**Figure 7:**
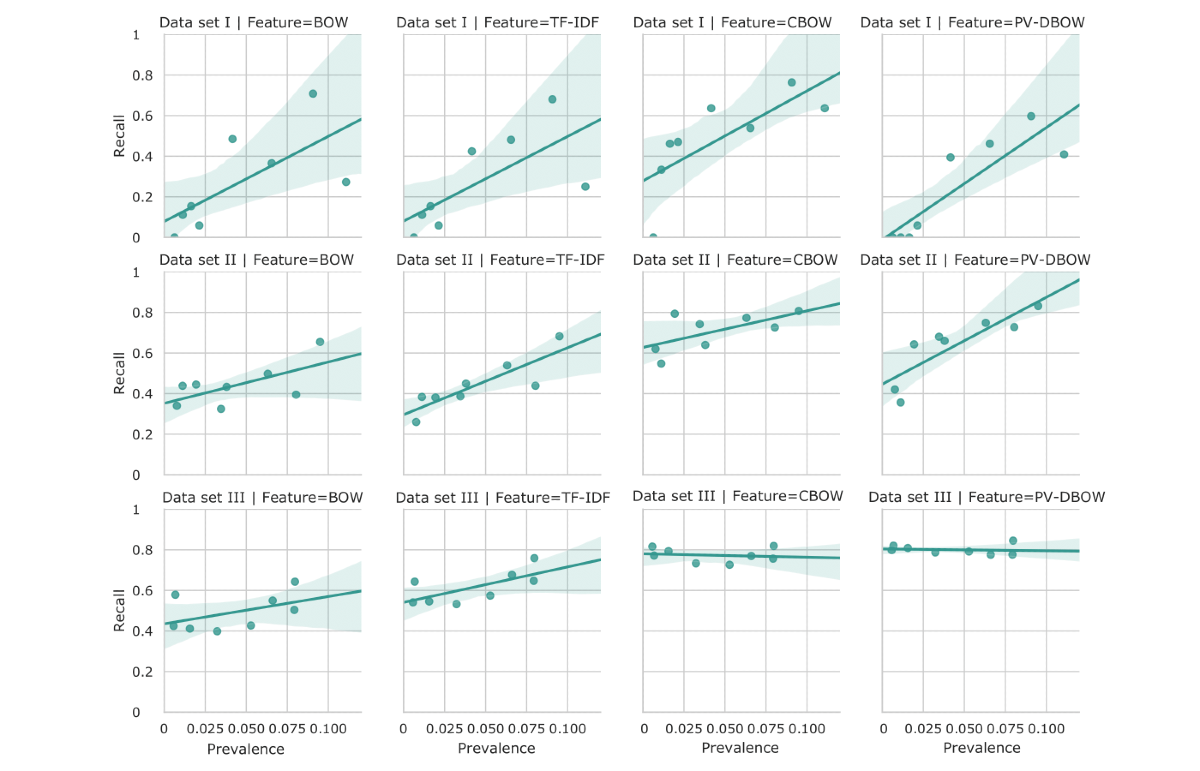
Recall scores as a function of the symptom prevalence in 3 considered data sets for all the features. BOW: bag-of-words; CBOW: continuous BOW; PV-BOW: paragraph vector–distributed BOW; TF-IDF: term frequency–inverse document frequency.

For all codes, the performance (area under the ROC [AUROC] curves and area under the precision-recall curves) of PV-DBOW features with logistic regression drastically improved with the size of the training set. For TF-IDF features also, there was a slight improvement, but it was less pronounced (Figure 6). More importantly, we observed that when increasing the size of the training set, the low prevalence of the symptoms does not affect the performance of embedding-based features (CBOW and PV-DBOW; Figure 7). Next, although the performance obtained with TF-IDF features was high, the computational performance was drastically affected by the increasing size of the training set. It takes 2 minutes and 1.6 GB of memory to train the model with PV-DBOW features, whereas the model with TF-IDF features requires 2.3 GB of memory and takes almost 3 hours (Table 2).

**Table 2:** Computational resources used for each classifier by feature type for data sets II and III.

Feature type and data set		Random forest		Logistic regression	
		Memory, MB	Run time, hours:minutes:seconds	Memory, MB	Run time, hours:minutes:seconds
**BOW^a^**					
	II	310	00:04:10	340	00:21:35
	III	3500	07:22:02	3400	23:17:20^b^
**TF-IDF^c^**					
	II	310	00:04:15	270	00:03:04
	III	3400	06:37:04	2300	02:47:30
**CBOW^d^**					
	II	193	00:03:02	180	00:01:17
	III	1700	01:21:11	1700	00:16:36
**PV-DBOW^e^**					
	II	170	00:03:35	89	00:00:34
	III	1100	01:41:18	1600	00:02:13

^a^BOW: bag-of-words.

^b^No convergence after 100,000 iterations.

^c^TF-IDF: term frequency–inverse document frequency.

^d^CBOW: continuous BOW.

^e^PV-DBOW: paragraph vector–distributed BOW.

### Enriching the Training Set With Weak Labels Enhances the Performance Further

The original cohort contained many notes that do not contain ICD-10 codes from the R chapter, leading to a substantial reduction in the number of notes available to train our model. Indeed, an additional 1,290,170 notes from the patients included in our cohort did not contain any ICD-10 code for symptoms.

To use these notes, they were processed using a weak supervision approach to determine the presence or absence of symptoms belonging to the R00-R09 categories. Then, the weakly labeled notes were added to data set D for training the classifier (ie, data set IV). For comparison, we also trained a model using only the weakly labeled notes (ie, data set V). Then, the 2 models were tested on test set III with ICD-10 codes for labels. The weak labeling model was also applied to the test set to extract weak labels for testing. Given the poor scaling performance of TF-IDF features compared with that of PV-DBOW, this experiment was performed solely with the PV-DBOW features.

**Figure 8:**
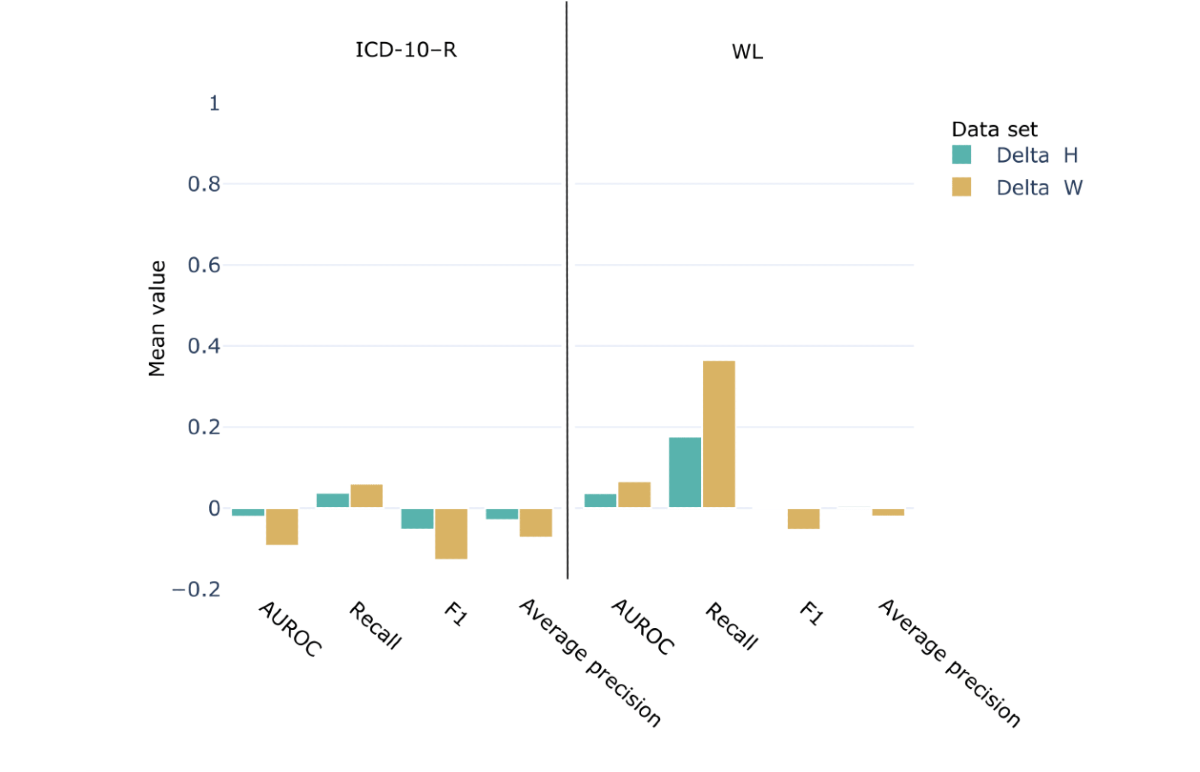
Performance metrics differential for the weak labeling experiment. Delta H represents the score difference between the hybrid data set IV and the baseline data set III (score [IV]–score [III]). Delta W represents the score difference between the weakly labeled data set V and the baseline data set III (score[V]–score [III]) The left panel shows the score calculated using International Classification of Disease–10th revision–R (ICD-10–R) codes for labels and the right panel shows the score calculated treating the weak labels (WL) as true labels in the test set. AUROC: area under the receiver operating characteristic curve.

Figure 8 shows the difference in performance between the enriched data set (IV) and the baseline data set (III). Overall, the recall score improved by 3.8%. However, the AUROC score was reduced by 2.1%. This decrease in the AUROC score can be attributed to the number of false-positive predictions. As the model was trained on mixed labels (ICD-10 and weak labels) but tested on ICD-10 codes, such increase in predictions flagged as false positives was expected. However, treating the weak labels as *true* labels for the test set led to an increase in recall score by 17.7% and an increase in AUROC score by 3.7%.

Use of only weakly labeled notes for training (data set V) and testing on ICD-10 labels led to a 6% increase in recall score and a 9.3% decrease in the AUROC score. Finally, using the weak labels as *true* labels for the test set, the weakly labeled notes performed 36.6% (recall) and 6.6% (AUROC) better than the baseline data set.

### Embedding-Based Features Perform Better Than TF-IDF Features on an External Validation Set

We selected a set of 56.65% (571/1008) notes from the i2b2 2009 challenge annotated for symptom extraction [52] containing mentions of symptoms of cough (R05), abnormalities of breathing (R06), and pain in throat and chest (R07). The logistic regression models trained on data set III using TF-IDF and PV-DBOW features were used to predict the presence of the 3 classes of symptoms.

**Figure 9:**
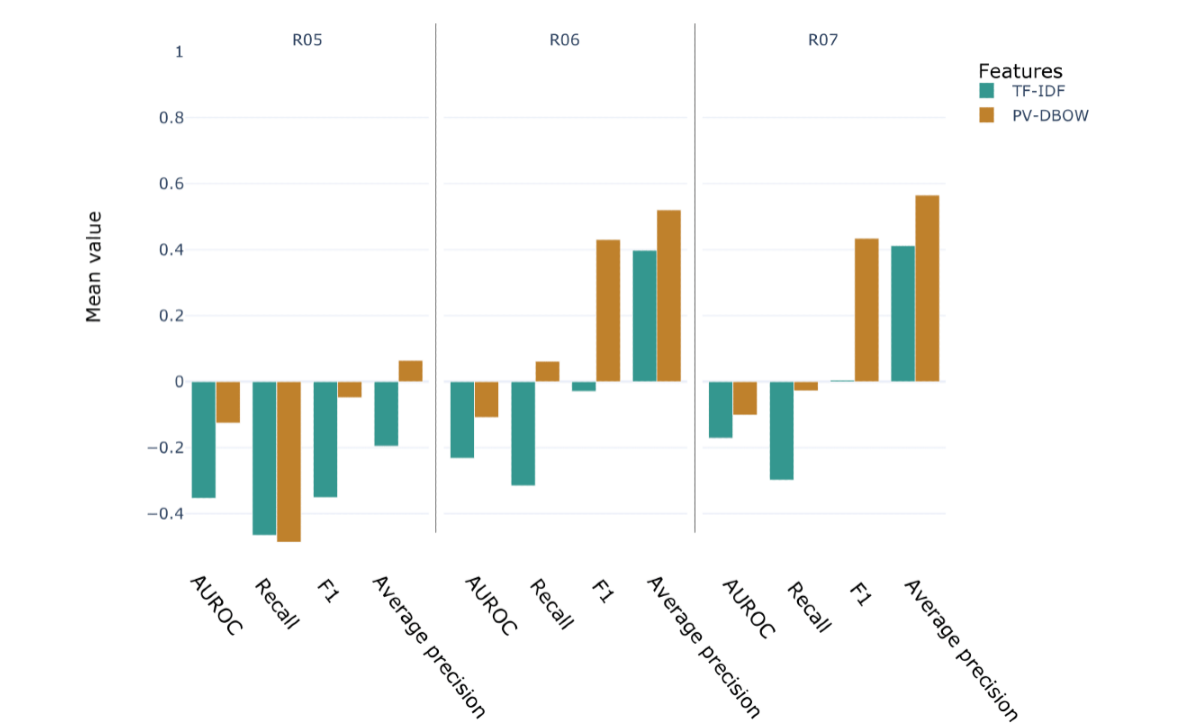
Performance metrics differential for the external validation set. The score has been calculated as the difference between the score obtained on the external validation set and the baseline data set III (score [Informatics for Integrating Biology and the Bedside]–score [Stanford Health Care]). Term frequency–inverse document frequency (TF-IDF) represents the logistic regression model trained with TF-IDF features. Paragraph vector–distributed bag-of-words (PV-DBOW) represents the logistic regression model trained with PV-DBOW features. International Classification of Disease–10th revision–R codes have been used as reference labels to compute the metrics. AUROC: area under the receiver operating characteristic curve.

Overall, the model trained with PV-DBOW features performed well when used to predict symptoms from the i2b2 notes. Figure 9 shows the difference in scores between the i2b2 data set and the baseline data set III trained using TF-IDF and PV-DBOW features for the set of 3 selected classes of symptoms. For R06 and R07, PV-DBOW, recall, and AUROC scores were within the range of the scores obtained when tested on the SHC notes. However, the F1 and average precision scores were >40 points better on the i2b2 notes. On the other hand, the model trained with TF-IDF features performed poorly. The recall and AUROC scores were 20 to 30 points lower than when tested on the SHC notes. The F1 score was similar to that obtained with the SHC notes. However, the average precision was almost 30 points higher than that of the SHC notes (Figure 9). For both PV-DBOW and TF-IDF features, the performance of the symptom *cough* decreased when tested on the i2b2 set compared with the SHC notes.

**Figure 10:**
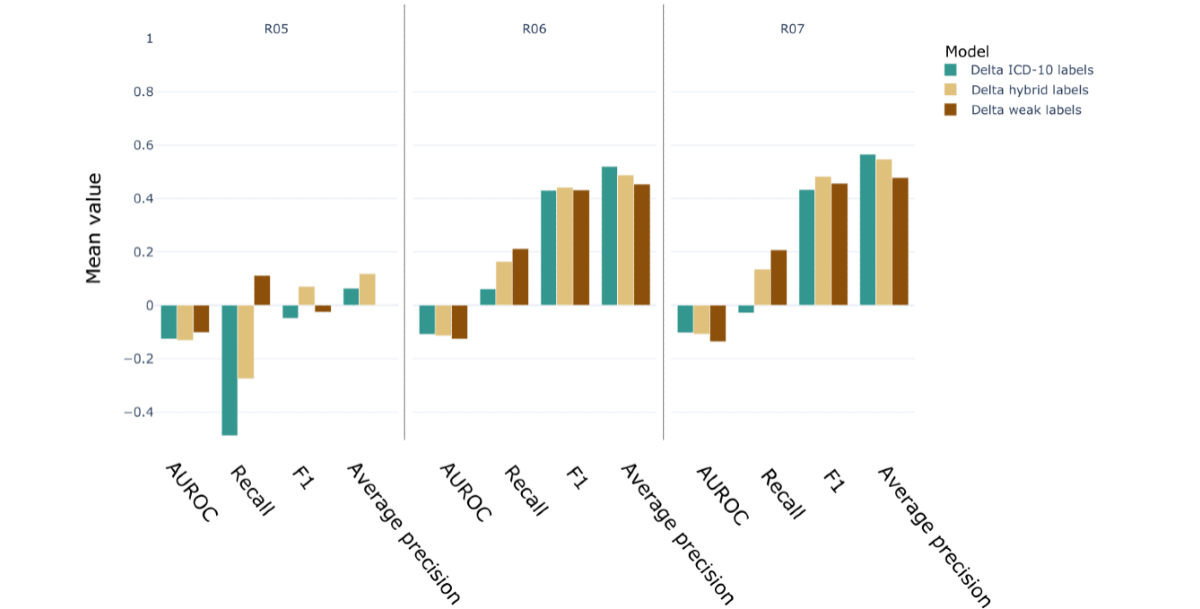
Performance metrics differential for the external validation set. The validation was performed for three models using paragraph vector–distributed bag-of-words features only, trained using different labels: International Classification of Disease–10th revision–R, the weak labels, and the hybrid labels. The score differences are computed relative to the baseline data set III (score [Informatics for Integrating Biology and the Bedside]–score [Stanford Health Care]). AUROC: area under the receiver operating characteristic curve.

Finally, the models trained with the hybrid labels and weak labels using the PV-DBOW features were also tested on the i2b2 notes. For both models, the recall and AUROC scores were within the range of those obtained with the SHC notes. However, the F1 and average precision scores were approximately 50 points higher than when tested with the SHC notes, reinforcing the conclusion that even though the models were trained on pseudolabels, they still perform well when tested on gold labels (Figure 10). Typically, recall performed better when hybrid or weak labels were used for training than when ICD-10 codes were used. Similar to the use of ICD-10 codes as labels, the performance for R05 decreased for the i2b2 notes.

### Analysis of Misclassified Cases

**Figure 11:**
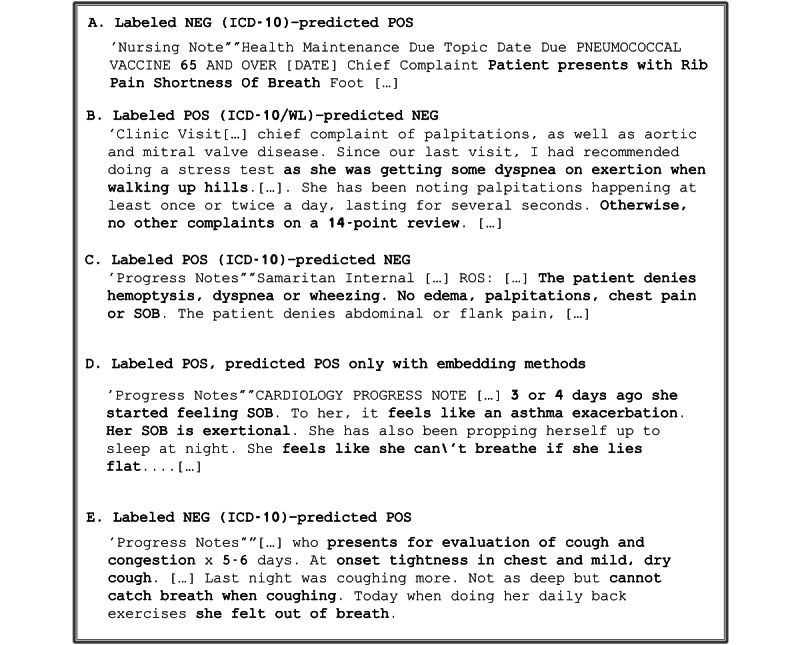
Snippet examples of mislabeled notes for R06 class of symptoms. ICD-10: International Classification of Disease–10th revision; NEG: negative; POS: positive; WL: weak labels.

To illustrate that despite the low quality of training labels used, the classification models were able to correctly classify notes, we show a few examples of the presence of abnormality of breathing symptoms in Figure 11. Snippets (A) and (E) show examples where the predictions were flagged as false positive but turned out to be true-positive cases. Snippets (B) and (C) show 2 examples that were flagged as false negative; however, when reading the note, the symptom was clearly absent (historical for (B) and negated for (C). Finally, snippet (D) shows an example that was correctly predicted only when embedding features were used.

## Discussion

### Principal Findings

We trained *one-versus-all* multi-label classification models using four featurization methods, namely BOW, TF-IDF, CBOW, and PV-DBOW, to predict the presence of signs and symptoms related to abnormalities in the circulatory and respiratory systems. The challenging lack of labels for training such models was addressed using 2 label extraction strategies. First, we extracted labels based on a subset of ICD-10 codes from EHR encounter data. This approach yielded good predictive performance, as evidenced by external validation. Relying on the coded part of EHR to extract training labels leaves a large part of progress notes untouched, as ICD-10 codes for symptoms are rarely used. The second approach we used was a method to extract training labels by leveraging clinical named entity recognition and a weak supervision pipeline. This approach not only allowed us to make use of a much larger set of notes for training but also significantly improved the predictive performance, both on an SHC test set and an external validation set.

Although TF-IDF features yielded the best performance overall (Figure 4), the size of the feature vector is the size of the corpus, leading rapidly to intractable size and computational inefficiency when the corpus size increased (Table 2), whereas embedding methods such as CBOW and PV-DBOW led to a fixed feature vector length, independent of the training corpus size. The main computing cost in such an approach lies in the pretraining of the embedding vectors, which must be performed only once. Training a classifier on any data set size led only to a minor increase in computational cost, making this approach more desirable.

Unfortunately, the results on a small training set were not satisfactory as these types of models are known to be extremely data hungry. The performance is expected to be more reasonable with larger data set sizes. We observed this in our experiments; when the training set size was increased, the performance also increased significantly. For example, the most notable performance improvement was observed for the recall, which increased from 0.25 to 0.8 for PV-DBOW features (Figure 4). This is important because when predicting the presence or absence of symptoms, minimizing the false-negative rate is desirable. Moreover, owing to the nature of our training labels, the absence of an ICD-10 code does not mean the absence of the symptom, whereas the presence of the code more likely signifies the presence of the symptom. Moreover, the effect of the low prevalence of some codes on the performance became negligible with increasing data set size and the use of PV-DBOW features, suggesting that the use of a resampling method is not necessary if training on larger data sets (Figure 6).

Next, enriching the largest data set with unlabeled notes using a weak supervision approach for labeling yielded an overall gain in performance. This result not only suggests that more is better but also points to the conclusion that the use of ICD-10 codes as labels to extract the presence of symptoms from clinical notes can be improved by using weak labeling pipelines to label previously unlabeled notes. Indeed, external validation of our models showed a large increase in performance of the PV-DBOW features. We attribute this gain to the quality of labels in the external validation data set, resulting in a drop in false-positive predictions. This experiment also suggests that although the quality of the labels used to train the models was not optimal, the model was still able to learn enough to reliably predict the presence of symptoms. On the other hand, the poor performance of the TF-IDF features suggests that the high performance observed on the SHC notes might be owing to overfitting of the features rather than a good predictive power. However, the increase in average precision suggests that the false-positive rate is reduced owing to the higher quality of the labels. Although TF-IDF seems to work well within one context, it is likely to fail when testing at other sites.

It is worth noting that the performance for cough symptoms (R05) decreased significantly when tested on our external validation data set. The causes for such a drop have not been investigated, but Figure 10 offers some hints about a labeling issue. Indeed, the recall score performed poorly when using the model trained with ICD-10 codes as labels but increased when using the weak labels as ground truth for training.

The automatic classification of clinical text into specific ICD codes is a common task, and various state-of-the-art models have been developed over the years. Although our objective is different, it is worth comparing our classification results with some of the available work. Moons et al [54] recently compared multiple state-of-the-art models for ICD coding of clinical records, using public data sets encoded with both ICD-9 (MIMIC-III [55]) and ICD-10 (CodiEsp [56]). They reported micro- and macro-F1, micro-AUROC, and Precision@5 for multiple subsets of MIMIC-III and CodiEsp using multiple deep learning architectures. As they did not report recall or the prevalence of each class, a direct comparison with our work is difficult. However, it is worth noting that the best-performing model on the MIMIC-III data set (using ICD-9 codes) yields a macro-F1 of 64.85. Their best-performing model on CodiEsp (using ICD-10 codes) yields a macro-F1 of 11.03. Our macro-F1 of 24.66 falls in between these values, suggesting that our performance lies within the range of some of the best-performing deep learning models available.

We note that although we are using a data set containing gold standard annotations, a direct comparison with previous results from Steinkamp et al [52] is not possible. Both experiments are fundamentally different. Our objective was to lay out strategies to generate training labels for a symptom classification task and demonstrate that if sufficient training data are provided, such strategies will yield good predictive performance. We did not aim to extract all symptoms from the notes or create new named entity recognition models. The use of the external data set, labeled by Steinkamp et al [52], was meant to show that (1) our models, although trained on SHC data, perform well on another institution’s data and, (2) considering that our models were trained on pseudolabels, they performed well on a test set containing gold labels.

Recent work has also seen the rise in transformers for NLP tasks. Although these methods are gaining popularity, the adaptation of such language model to the clinical use case is not straightforward. First, transformer models usually have a relatively short fixed maximum input length (eg, 412 tokens for bidirectional encoder representations from transformers [BERT]–based models). Clinical notes in general, and progress notes in particular, tend to be much longer than that (eg, in our case, the note length is closer to a couple of thousands of tokens). Moreover, transformer-based models trained on open domain text are not suitable for clinical text and must be fine-tuned to maximize performance. Although some BERT adaptations for the clinical domain have been released recently (eg, ClinicalBERT [57], BioBERT [58], or BlueBERT [59]), these publicly available models might not be suitable for the task at hand. Reasons why BERT-based models might not be suitable include attention dilution and the use of subword tokenization rather than word-level tokenization [60]. Finally, finding the best embedding method for note classification was outside the scope of our study. For these reasons, we did not include transformers in our comparison.

### Conclusions

In this study, we introduced 2 methods to extract labels from EHR data sets for the training of a classifier for clinical notes. Multiple featurization methods were investigated, showing that PV-DBOW is clearly superior in terms of transferability and scaling. Although the use of ICD-10 codes present in the encounter data is a simple way of extracting training labels, the poor accuracy of the coding leads to less accurate models. Using a weak labeling pipeline to extract such labels yields improved performance and allows for the use of more notes as we are not relying on the presence of codes. Both approaches have been validated with an external set of notes containing gold labels, which showed the superiority of the weak labeling approach. Using ICD-10 codes for initial labels, we grouped a wide variety of signs and symptoms under the same label, learning classes of symptoms rather than specific symptoms. For example, R06 (abnormalities of breathing) covers a variety of breathing abnormalities; for example, dyspnea, wheezing, or hyperventilation. Such granularity in the symptoms is beyond the scope of this study and thus has not been investigated. However, the good performance of the weak labeling pipeline suggests that such an approach to generate more granular labels (eg, to distinguish between wheezing and shortness of breath in the R06 category) could be used. Moreover, the nature of the *one-versus-all *approach allows us to add a new category without having to retrain our model on all labels. Finally, the good performance and computational efficiency of the PV-DBOW features with logistic regression model would make such an expansion of the model computationally cheap.

## Appendix

Multimedia Appendix 1 Supplementary tables.

## Abbreviations

AUROC: area under the receiver operating characteristic curve
BERT: bidirectional encoder representations from transformers
BOW: bag-of-words
CBOW: continuous bag-of-words
EHR: electronic health record
ICD-10: International Classification of Disease–10th revision
i2b2: Informatics for Integrating Biology and the Bedside
MIMIC: Medical Information Mart for Intensive Care
NLP: natural language processing
PV-DBOW: paragraph vector–distributed bag-of-words
SHC: Stanford Health Care
TF-IDF: term frequency–inverse document frequency

## Data and Code Availability

Protected Health Information restrictions apply to the availability of the Stanford Health Care clinical data set presented here, which were used under institutional review board approval for use only in this study and thus are not publicly available. The code can be made available upon request.
