# Supplementary material for: Strategies to Address the Lack of Labeled Data for Supervised Machine Learning Training With Electronic Health Records: Case Study for the Extraction of Symptoms From Clinical Notes
Source: JMIR Med Inform. 2022 Mar 14;10(3):e32903. doi: 10.2196/32903 (PMC8961340; doi:10.2196/32903)
Supplement: Multimedia Appendix 1 [file medinform_v10i3e32903_app1.docx]

# Strategies to address the lack of labelled data for supervised machine learning training with electronic health records: a case study for extraction of symptoms from clinical notes – Supplementary Material

## Methods – modeling

The following parameters were used in the modeling part:

Random Forest: n_estimators=100, class_weight=balanced, threshold=0.5

Logistic Regression: L2 regularization, C=30, solver=lbfgs, class_weight=balanced, threshold=0.5

Table S1 Prevalence of R codes in the considered datasets

| Sign or symptom category | Dataset I | Dataset II | Dataset III |
| --- | --- | --- | --- |
| Abnormalities of heartbeat (R00) |  |  |  |
| Train set | 4.64% | 5.34% | 4.98% |
| Validation set | 5.11% | 3.67% | 4.47% |
| Test set | 6.55% | 3.80% | 5.28% |
| Cardiac murmurs and other cardiac sounds (R01) |  |  |  |
| Train set | 1.01% | 0.59% | 0.56% |
| Validation set | 0.57% | 0.55% | 0.64% |
| Test set | 0.63% | 0.77% | 0.57% |
| Abnormal blood pressure reading, without diagnosis (R03) |  |  |  |
| Train set | 1.29% | 1.51% | 1.55% |
| Validation set | 0.85% | 1.42% | 1.36% |
| Test set | 1.64% | 1.94% | 1.56% |
| Hemorrhage from respiratory passages (R04) |  |  |  |
| Train set | 0.89% | 0.65% | 0.76% |
| Validation set | 0.71% | 1.07% | 0.76% |
| Test set | 1.13% | 1.12% | 0.68% |
| Cough (R05) |  |  |  |
| Train set | 7.38% | 6.99% | 7.82% |
| Validation set | 9.80% | 7.23% | 7.71% |
| Test set | 9.07% | 9.50% | 7.97% |
| Abnormalities of breathing (R06) |  |  |  |
| Train set | 6.89% | 7.39% | 7.93% |
| Validation set | 6.11% | 6.93% | 7.73% |
| Test set | 11.08% | 8.04% | 8.08% |
| Pain in throat and chest (R07) |  |  |  |
| Train set | 5.20% | 6.17% | 6.61% |
| Validation set | 6.25% | 6.27% | 6.31% |
| Test set | 4.16% | 6.31% | 6.10% |
| Other symptoms and signs involving the circulatory and respiratory system (R09) |  |  |  |
| Train set | 2.90% | 2.85% | 3.08% |
| Validation set | 2.41% | 3.55% | 3.12% |
| Test set | 2.14% | 3.46% | 3.23% |

Table S2 ICD10-CM codes for signs and symptoms for the circulatory and respiratory systems and corresponding UMLS CUIs

| ICD10-CM code | List of UMLS CUI |
| --- | --- |
| R00 | C0030252, C0039231, C0085610, C0232190, C0340468, C0428977, C0476258, C0478110, C0694459, C1744601, C2910380, C2910381, C2910382 |
| R01 | C0018808, C0232255, C0232257, C0476263, C0478112, C0495661, C0495662, C0850071, C0866959 |
| R03 | C0392682, C0476454, C0495664 |
| R04 | C0014591, C0019079, C0151701, C0476274, C0478113, C0478116, C0576995, C1390055, C2958656, C2977671 |
| R05 | C0010200 |
| R06 | C0003578, C0008039, C0013404, C0019521, C0020578, C0026635, C0037383, C0037384, C0038450, C0043144, C0085619, C0231835, C0425481, C0476287, C0748355, C1260922, C1313952, C2910383, C2910384, C2910385, C2910386 |
| R07 | C0008031, C0008033, C0029537, C0232286, C0242429, C0423729, C0476278, C0495666, C0877023 |
| R09 | C0004044, C0027424, C0032231, C0032781, C0034642, C0159054, C0162297, C0232112, C0232132, C0235567, C0476275, C0476276, C0476277, C0476283, C0476284, C0476285, C0478110, C0478115, C0495668, C0546947, C0700292, C1444565, C1561822, C1955514 |

Table S3 Input vector size for the training set according to the vectorization method

| Feature type | Dataset I | Dataset II | Dataset III |
| --- | --- | --- | --- |
| BOW | (2480, 20281) | (20500, 53637) | (326934, 225595) |
| TFIDF | (2480, 20281) | (20500, 53637) | (326934, 225595) |
| CBOW | (2480, 200) | (20500, 200) | (326934, 200) |
| PV-DBOW | (2480, 300) | (20500, 300) | (326934, 300) |

Table S4 Computational resources used for each classifier by feature type for dataset II

| Feature type | RF |  | LR |  |
| --- | --- | --- | --- | --- |
| BOW | 310MB | 00:04:10 | 340MB | 00:21:35 |
| TFIDF | 310MB | 00:04:15 | 270MB | 00:03:04 |
| CBOW | 193MB | 00:03:02 | 180MB | 00:01:17 |
| PV-DBOW | 170MB | 00:03:35 | 89MB | 00:00:34 |

Table S5 Computational resources used for each classifier by feature type for dataset III.
* No convergence after 100,000 iterations

| Feature type | RF |  | LR |  |
| --- | --- | --- | --- | --- |
| BOW | 3.5GB | 07:22:02 | 3.4GB | 23:17:20* |
| TFIDF | 3.4GB | 06:37:04 | 2.3GB | 02:47:30 |
| CBOW | 1.7GB | 01:21:11 | 1.7GB | 00:16:36 |
| PV-DBOW | 1.1GB | 01:41:18 | 1.6GB | 00:02:13 |

Table S6 Recall scores for Random Forest and Logistic Regression classifiers averaged over all considered codes

|  | **RF** | | | | **LR** | | | |
| --- | --- | --- | --- | --- | --- | --- | --- | --- |
|  | **Recall score** | | | | **Recall score** | | | |
| **Model** | **Mean** | **Min** | **Max** | **SD** | **Mean** | **Min** | **Max** | **SD** |
| **BOW** |  |  |  |  |  |  |  |  |
| Dataset I | 0.01 | 0.00 | 0.10 | 0.03 | 0.27 | 0.00 | 0.71 | 0.24 |
| Dataset II | 0.03 | 0.00 | 0.13 | 0.04 | 0.44 | 0.32 | 0.65 | 0.10 |
| Dataset III | 0.07 | 0.01 | 0.22 | 0.07 | 0.49 | 0.40 | 0.64 | 0.09 |
| **TFIDF** |  |  |  |  |  |  |  |  |
| Dataset I | 0.002 | 0.00 | 0.01 | 0.005 | 0.27 | 0.00 | 0.68 | 0.24 |
| Dataset II | 0.02 | 0.00 | 0.07 | 0.02 | 0.44 | 0.26 | 0.68 | 0.26 |
| Dataset III | 0.06 | 0.00 | 0.17 | 0.06 | 0.61 | 0.53 | 0.76 | 0.08 |
| **CBOW** |  |  |  |  |  |  |  |  |
| Dataset I | 0.03 | 0.00 | 0.24 | 0.08 | 0.48 | 0.00 | 0.76 | 0.23 |
| Dataset II | 0.05 | 0.00 | 0.22 | 0.07 | 0.71 | 0.55 | 0.81 | 0.09 |
| Dataset III | 0.09 | 0.00 | 0.28 | 0.09 | 0.77 | 0.73 | 0.82 | 0.03 |
| **PV-DBOW** |  |  |  |  |  |  |  |  |
| Dataset I | 0.00 | 0.00 | 0.00 | 0.00 | 0.24 | 0.00 | 0.60 | 0.25 |
| Dataset II | 0.001 | 0.00 | 0.01 | 0.002 | 0.63 | 0.36 | 0.83 | 0.16 |
| Dataset III | 0.02 | 0.00 | 0.06 | 0.02 | 0.80 | 0.78 | 0.85 | 0.02 |

Table S7 Performance metrics for all classes of symptoms (R00-R09) for dataset III enriched with weakly labeled data tested on ICD10-CM codes as labels

| Sign or symptom category | AUROC | Recall | F1 | Average precision |
| --- | --- | --- | --- | --- |
| R00 |  |  |  |  |
| Dataset III | 0.883 | 0.791 | 0.330 | 0.176 |
| Dataset IV | 0.873 | 0.853 | 0.263 | 0.140 |
| Dataset V | 0.808 | 0.898 | 0.161 | 0.085 |
| R01 |  |  |  |  |
| Dataset III | 0.884 | 0.798 | 0.048 | 0.021 |
| Dataset IV | 0.842 | 0.817 | 0.031 | 0.014 |
| Dataset V | 0.757 | 0.809 | 0.020 | 0.009 |
| R03 |  |  |  |  |
| Dataset III | 0.901 | 0.809 | 0.137 | 0.063 |
| Dataset IV | 0.893 | 0.827 | 0.123 | 0.057 |
| Dataset V | n/a | n/a | n/a | n/a |
| R04 |  |  |  |  |
| Dataset III | 0.918 | 0.820 | 0.081 | 0.036 |
| Dataset IV | 0.853 | 0.795 | 0.040 | 0.018 |
| Dataset V | 0.809 | 0.866 | 0.024 | 0.011 |
| R05 |  |  |  |  |
| Dataset III | 0.923 | 0.845 | 0.507 | 0.318 |
| Dataset IV | 0.917 | 0.889 | 0.413 | 0.248 |
| Dataset V | 0.894 | 0.935 | 0.253 | 0.142 |
| R06 |  |  |  |  |
| Dataset III | 0.850 | 0.776 | 0.351 | 0.194 |
| Dataset IV | 0.841 | 0.846 | 0.305 | 0.169 |
| Dataset V | 0.740 | 0.838 | 0.213 | 0.115 |
| R07 |  |  |  |  |
| Dataset III | 0.868 | 0.776 | 0.328 | 0.175 |
| Dataset IV | 0.862 | 0.847 | 0.251 | 0.134 |
| Dataset V | 0.754 | 0.868 | 0.162 | 0.085 |
| R09 |  |  |  |  |
| Dataset III | 0.856 | 0.787 | 0.191 | 0.092 |
| Dataset IV | 0.848 | 0.822 | 0.165 | 0.081 |
| Dataset V | 0.771 | 0.801 | 0.110 | 0.054 |

Table S8 Performance metrics for all classes of symptoms (R00-R09) for dataset III enriched with weakly labeled data tested on weak labels

| Sign or symptom category | AUROC | Recall | F1 | Average precision |
| --- | --- | --- | --- | --- |
| R00 |  |  |  |  |
| Dataset III | 0.798 | 0.603 | 0.314 | 0.156 |
| Dataset IV | 0.815 | 0.738 | 0.289 | 0.151 |
| Dataset V | 0.832 | 0.923 | 0.215 | 0.118 |
| R01 |  |  |  |  |
| Dataset III | 0.788 | 0.537 | 0.236 | 0.105 |
| Dataset IV | 0.867 | 0.822 | 0.244 | 0.127 |
| Dataset V | 0.892 | 0.919 | 0.187 | 0.100 |
| R04 |  |  |  |  |
| Dataset III | 0.671 | 0.287 | 0.200 | 0.094 |
| Dataset IV | 0.728 | 0.561 | 0.237 | 0.115 |
| Dataset V | 0.797 | 0.870 | 0.217 | 0.117 |
| R05 |  |  |  |  |
| Dataset III | 0.835 | 0.618 | 0.524 | 0.333 |
| Dataset IV | 0.841 | 0.725 | 0.496 | 0.311 |
| Dataset V | 0.856 | 0.914 | 0.388 | 0.237 |
| R06 |  |  |  |  |
| Dataset III | 0.699 | 0.528 | 0.262 | 0.135 |
| Dataset IV | 0.723 | 0.665 | 0.267 | 0.142 |
| Dataset V | 0.760 | 0.873 | 0.246 | 0.137 |
| R07 |  |  |  |  |
| Dataset III | 0.707 | 0.490 | 0.311 | 0.166 |
| Dataset IV | 0.750 | 0.673 | 0.315 | 0.173 |
| Dataset V | 0.777 | 0.905 | 0.276 | 0.158 |
| R09 |  |  |  |  |
| Dataset III | 0.821 | 0.710 | 0.217 | 0.103 |
| Dataset IV | 0.852 | 0.820 | 0.208 | 0.106 |
| Dataset V | 0.867 | 0.924 | 0.163 | 0.086 |

Table S9 Benchmark N2C2: PV-DBOW model trained with datasets III, IV and V

| Sign or symptom category | AUROC | Recall | F1 | Average precision |
| --- | --- | --- | --- | --- |
| R05 |  |  |  |  |
| Dataset III | 0.797 | 0.358 | 0.458 | 0.382 |
| Dataset IV | 0.792 | 0.569 | 0.578 | 0.437 |
| Dataset V | 0.821 | 0.956 | 0.481 | 0.318 |
| R06 |  |  |  |  |
| Dataset III | 0.742 | 0.840 | 0.782 | 0.715 |
| Dataset IV | 0.735 | 0.941 | 0.793 | 0.682 |
| Dataset V | 0.724 | 0.989 | 0.783 | 0.647 |
| R07 |  |  |  |  |
| Dataset III | 0.765 | 0.747 | 0.762 | 0.740 |
| Dataset IV | 0.760 | 0.911 | 0.812 | 0.723 |
| Dataset V | 0.732 | 0.983 | 0.786 | 0.654 |

Table S10 Benchmark N2C2: TFIDF model trained on dataset III

| Sign or symptom category | AUROC | Recall | F1 | Average precision |
| --- | --- | --- | --- | --- |
| R05 |  |  |  |  |
| Dataset III | 0.578 | 0.292 | 0.307 | 0.264 |
| R06 |  |  |  |  |
| Dataset III | 0.641 | 0.335 | 0.470 | 0.651 |
| R07 |  |  |  |  |
| Dataset III | 0.414 | 0.139 | 0.129 | 0.223 |
